# Synthesis of α-Fe_2_O_3_ and Fe-Mn Oxide Foams with Highly Tunable Magnetic Properties by the Replication Method from Polyurethane Templates

**DOI:** 10.3390/ma11020280

**Published:** 2018-02-11

**Authors:** Yuping Feng, Jordina Fornell, Huiyan Zhang, Pau Solsona, Maria Dolors Barό, Santiago Suriñach, Eva Pellicer, Jordi Sort

**Affiliations:** 1 Departament de Física, Universitat Autònoma de Barcelona, E-08193 Bellaterra, Cerdanyola del Vallès, Spain; fengyupingcsu@gmail.com (Y.F.); abuzhang1984@gmail.com (H.Z.); Pau.Solsona@uab.cat (P.S.); dolors.baro@uab.es (M.D.B.); Santiago.Surinyach@uab.cat (S.S.); eva.pellicer@uab.cat (E.P.); jordi.sort@uab.cat (J.S.); 2 State Key Laboratory of Optoelectronic Materials and Technologies, School of Electronics and Information Technology, Sun Yat-Sen University, Guangzhou 510275, China; 3 Institució Catalana de Recerca i Estudis Avançats (ICREA), Pg. Lluís Companys 23, E-08010 Barcelona, Spain

**Keywords:** porous inorganic oxide foams, manganese ferrite, Iron oxide, replication processing, magnetic properties

## Abstract

Open cell foams consisting of Fe and Fe-Mn oxides are prepared from metallic Fe and Mn powder precursors by the replication method using porous polyurethane (PU) templates. First, reticulated PU templates are coated by slurry impregnation. The templates are then thermally removed at 260 °C and the debinded powders are sintered at 1000 °C under N_2_ atmosphere. The morphology, structure, and magnetic properties are studied by scanning electron microscopy, X-ray diffraction and vibrating sample magnetometry, respectively. The obtained Fe and Fe-Mn oxide foams possess both high surface area and homogeneous open-cell structure. Hematite (α-Fe_2_O_3_) foams are obtained from the metallic iron slurry independently of the N_2_ flow. In contrast, the microstructure of the FeMn-based oxide foams can be tailored by adjusting the N_2_ flow. While the main phases for a N_2_ flow rate of 180 L/h are α-Fe_2_O_3_ and FeMnO_3_, the predominant phase for high N_2_ flow rates (e.g., 650 L/h) is Fe_2_MnO_4_. Accordingly, a linear magnetization versus field behavior is observed for the hematite foams, while clear hysteresis loops are obtained for the Fe_2_MnO_4_ foams. Actually, the saturation magnetization of the foams containing Mn increases from 5 emu/g to 52 emu/g when the N_2_ flow rate (i.e., the amount of Fe_2_MnO_4_) is increased. The obtained foams are appealing for a wide range of applications, such as electromagnetic absorbers, catalysts supports, thermal and acoustic insulation systems or wirelessly magnetically-guided porous objects in fluids.

## 1. Introduction

Reticulated sponges made of polyurethane (PU) have been commercially available since the 1950s [1]. The first attempts to transfer porous templates into ceramic (i.e., oxide) foams by the powder slurry replication method date from early 1960s [2]. At present, the method has become widely available for many applications involving porous oxide materials: electromagnetic wave absorbers, gas sensors, catalysts, oil-water separators and lithium-ion batteries [3,4,5,6,7,8]. As a consequence, the polymeric sponge replication process has consolidated as a promising technique to create cellular oxide structures with 3D interconnected pores, characterized by high strength and high corrosion resistance in acid and alkaline media [9,10,11]. In the 1970s, metallic foams also started to be prepared by this method and they found applications such as battery electrodes, catalysts or ﬁlters [12,13,14]. Over the past ten years, the replication method has been extended to manufacture porous steels [15,16,17,18] and porous Cu-based [19] and Ti-based alloys [20,21].

While many studies have focused on metallic foams [15,16,17,18,19,20,21], fewer efforts have been devoted to synthesizing inorganic oxide foams through the polymer sponge replication process [22]. So far, to the best of our knowledge, the possibility to use this technique to produce oxide foams with magnetic properties has not been explored. 

Manganese ferrite (MnFe_2_O_4_) is a well-known ceramic compound with electrically insulating and soft ferrimagnetic properties at room temperature. It has been widely used in the electronics industry to fabricate magnetic cores for read/write heads for high-speed tape or hard disk recording [23,24]. More recently, MnFe_2_O_4_ has emerged as a promising material to be used as a catalyst [25,26], in hydrogen production technologies [24] or for oil-water separation [7]. Diverse preparation techniques, such as high energy ball milling [24,27], co-precipitation [28,29] or sol-gel routes [30], have been used to obtain MnFe_2_O_4_ powders and nanoparticles. However, the preparation of porous MnFe_2_O_4_ remains challenging and rather elusive. 

Meanwhile, magnetic foams made of Fe_2_O_3_, Fe_3_O_4_, Co or Ni are appealing since they combine the intrinsic properties of magnetic materials with the aforementioned advantages of the porous structures, constituting magnetic materials with high surface area, low density and high strength-to-weight ratio. For instance, ultralight Fe_2_O_3_/C foams produced using polyelectrolyte-grafted PU sponges [7] exhibit one of the highest oil-absorption capacities among the reported counterparts. Magnetic Fe_3_O_4_ nanoparticles/PU composites produced by in-situ blending methods have also been reported as good candidates for wastewater treatments, acting as carriers for immobilized microorganisms [30].

Therefore, the aim of the present work is to use the replication method to produce Fe and Fe-Mn oxides open-cell porous structures. The magnetic behavior of the foams is tuned, first, by the composition of the metallic slurry precursor and, secondly, by adjusting the N_2_ flow. The influence of the N_2_ flow and the Mn content, as well as the synergistic effect of porosity and magnetism are investigated.

## 2. Materials and Methods

Commercial Fe (97%) and Mn (99%) powders were used to coat a reticulated PU sponge by the impregnation method. First, the powders were mixed and mechanically milled in a planetary mill device (Fritsch Pulverisette 5, Fritsch, Idar-Oberstein, Germany) to reduce the powder size and to obtain the targeted composition, Fe or Fe-30Mn (Fe:Mn ratio of 70:30 nominal wt %). The raw powders were milled under Ar atmosphere with a ball-to-powder weight ratio of 10:1 at 300 rpm for 15 h. The particle size of the as-milled powders, calculated from a Scanning Electron Microscopy (SEM) image using the ImageJ software, was 10.8 ± 1.4 mm. To prepare the slurry, the milled powders were mixed with poly(ethylene glycol) (PEG) and distilled water. PEG acted as a binder and was used to control the slurry viscosity and to favor the adhesion of the powder particles to the sponge before sintering. The composition of the slurry is listed in Table 1. Subsequently, 1 cm^3^ of a commercial reticulated PU sponge, acting as organic template, was immersed into the slurry for 5 min to allow complete impregnation. The sponge was removed from the suspension and was squeezed to ensure that only a thin layer of slurry covered the skeleton of the PU template without blocking the pores. Then, the impregnated template was dried at room temperature for at least 12 h before sintering. Finally, the template was thermally removed at 260 °C and the debinded powders were sintered in a tubular furnace (Carbolite MTF 9/15, Parsons Lane, UK) at 1000 °C for 2 h under N_2_ flow ranging from 180 to 650 L/h.

SEM observations were done on a Zeiss Merlin microscope (Carl Zeiss Microscopy, Jena, Germany) equipped with an energy dispersive X-ray (EDX) spectroscopy detector (Oxford Instruments, Abingdon, UK) for compositional analyses. X-ray diffraction (XRD) was carried out on a Philips X’Pert diffractometer (Philips, Amsterdam, The Netherlands) using Cu K_α_ radiation. The measurements were performed in the angular range 2θ = 30–100° with a step size of 0.026°. Furthermore, Rietveld refinement of the XRD patterns using the X’Pert HighScore Plus software (PANalytical, Almelo, The Netherlands) was carried out to determine the cell parameters and the percentage of the constituent phases.

Hysteresis loops were recorded at room temperature using a vibrating sample magnetometer (VSM) from MicroSense (LOT-QuantumDesign, Darmstadt, Germany), with a maximum applied magnetic field of 20 kOe.

## 3. Results and Discussion

### 3.1. Microstructure and Compositional Analyses

**Figure 1:**
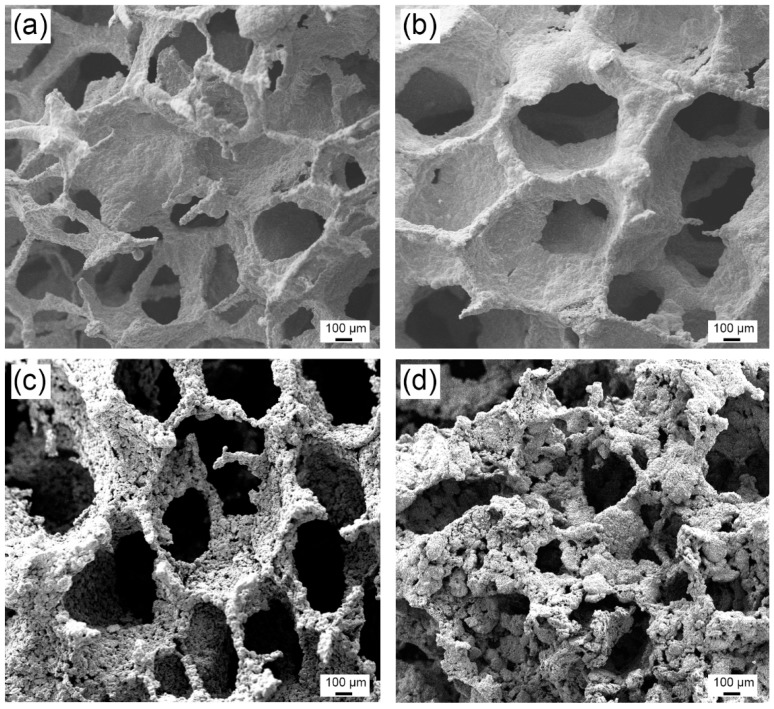
(**a**) SEM image of a Fe-O foam prepared at a nitrogen flow rate of 180 L/min. SEM image of Fe-Mn-O foams prepared under a nitrogen flow rate of (**b**) 180 L/h, (**c**) 350 L/h and (**d**) 650 L/h.

Figure 1 shows SEM images of the sintered open cell foams obtained by the replication process from Fe- (Figure 1a) and FeMn- (Figure 1b–d) containing slurries. At a first glance, a rather homogeneous pore distribution can be observed, with pore sizes of the order of 400 μm. Fully-compact pore walls were observed in the foams produced from the Fe- and FeMn-containing slurries obtained at the lowest N_2_ flow (Figure 1a,b); however, at larger N_2_ flows, the pore walls exhibit a nanoporous morphology although the distribution and the size of the big pores was not compromised. The composition of the foams obtained at different nitrogen flow rates, measured by energy-dispersive X-ray (EDX) analyses, is listed in Table 2. An O/Fe ratio of 1.56 is calculated for the foam produced from the Fe-containing slurry, suggesting the formation of Fe_2_O_3_. It is worth mentioning that no changes were observed in the foams produced under larger N_2_ flow (such as 350 L/h and 650 L/h) from the Fe-containing slurry in terms of pore morphology, integrity of the foam or chemical composition (not shown in the manuscript). However, the foams produced at open atmosphere were more powdery and brittle. In contrast, compositional changes were observed in the foams produced from the FeMn-containing slurry when changing the N_2_ flow. For the foam produced at 180 L/h, the O/Fe(Mn) ratio is 1.5 suggesting the formation of Fe_2_O_3_ or FeMnO_3_. Conversely, the O/Fe(Mn) ratio decreases to 1.38 and 1.27 as the N_2_ flow is increased to 350 L/h and 650 L/h, respectively, hence suggesting the formation of Fe_3−x_(Mn)_x_O_4_.

**Figure 2:**
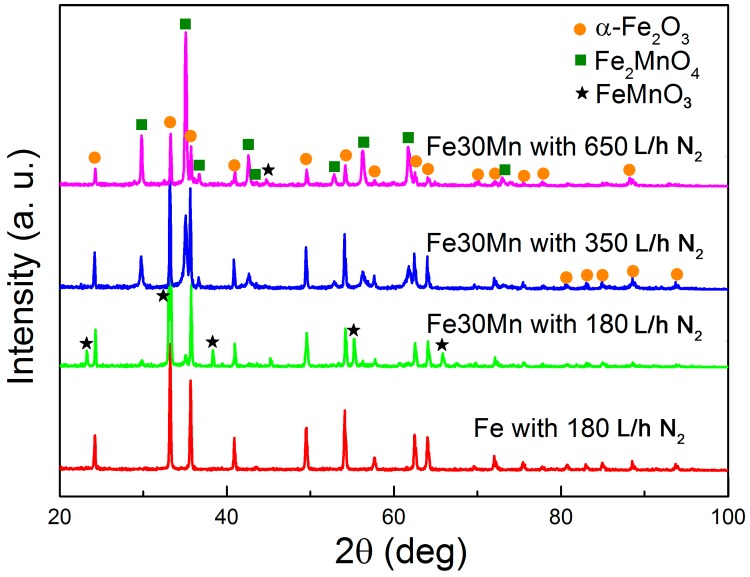
X-ray diffraction (XRD) patterns of the Fe-O foam produced at N_2_ flow of 180 L/h and Fe-Mn-O foams produced at a N_2_ flow of 180 L/h, 350 L/h and 650 L/h.

To shed light on the microstructure of the open-cell foams X-ray diffraction was carried out (Figure 2). As suggested by EDX analyses, the foams produced from the Fe-containing slurry consist of α-Fe_2_O_3_. In contrast, the XRD of the foams produced from the FeMn-containing slurry indicate a mixture of α-Fe_2_O_3_, FeMnO_3_ and Fe_2_MnO_4_ phases. Phase contributions and cell parameters estimated by Rietveld refinement are listed in Table 3. Sintering the FeMn-containing foams at higher N_2_ flow results in larger amounts of Fe_2_MnO_4_. As can be observed in Table 3, the amount of Fe_2_MnO_4_ increases from 8.5 to 74% when increasing the N_2_ flow from 180 to 650 L/h. Accordingly, the Fe_2_O_3_ phase percentage decreases from 60 to 26%. FeMnO_3_ only forms at a N_2_ flow of 180 L/h. 

### 3.2. Magnetic Properties

**Figure 3:**
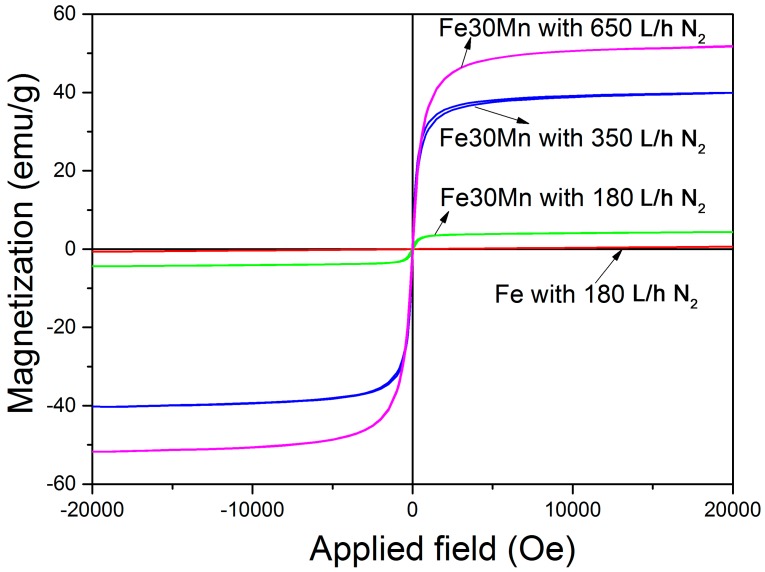
Room temperature hysteresis loops of four kinds of open-cell porous foams produced from the Fe- and FeMn-containing slurries at different nitrogen flow rate.

Figure 3 shows the hysteresis loops of the open-cell porous foams produced from the Fe- and FeMn-containing slurries at different nitrogen flow rates. A linear magnetization versus field behavior (reaching 0.6 emu/g for an applied field of 20 kOe) was observed in the Fe-O foams, in agreement with the reported behavior for antiferromagnetic α-Fe_2_O_3_. Clear hysteresis loops are observed in the FeMn-containing foams as a result of the ferrimagnetic character of the Fe_2_MnO_4_ phase, which exhibits a theoretical saturation magnetization, M_s_, of 77 emu/g at room temperature [31]. The FeMnO_3_ phase is known to exhibit a weakly ferrimagnetic response with M_s_ ~ 0.23 emu/g [32]. Accordingly, the saturation magnetization of the FeMn-containing foams ranges from 5 emu/g to 52 emu/g depending on the N_2_ flow rate, i.e., the relative amount of FeMnO_3_ and Fe_2_MnO_4_ phases. For instance, an M_s_ value of 37.7 emu/g would be expected for the foam produced under a N_2_ flow of 350 L/h as it contains 49% of Fe_2_MnO_4_. The observed M_s_ in this case (40 emu/g) is in quite good agreement with the expected value. The foam produced under a N_2_ flow of 650 L/h has M_s_ = 52 emu/g which is also in good agreement with the theoretically calculated value (57 emu/g).

## 4. Conclusions

Highly porous foams with homogeneous open-cell structure have been obtained by the replication process using polyurethane templates. The foams produced from the Fe-containing slurry consist of α-Fe_2_O_3_, independently of the N_2_ flow. In contrast, the phase composition of the foams produced from the FeMn-containing slurry can be tuned by adjusting the N_2_ flow. The main phases of the Fe-Mn-O foams sintered under a N_2_ flow of 180 L/h are α-Fe_2_O_3_ and FeMnO_3_ with minor content of Fe_2_MnO_4_. Increasing the N_2_ flow to 350 L/h results in a mixture of α-Fe_2_O_3_ and Fe_2_MnO_4_. The amount of manganese ferrite is further increased at a flow rate of 650 L/h. Hence, addition of Mn as well as the adjustment of the N_2_ flow allows tailoring the magnetic response of the foams from practically non-magnetic to soft ferrimagnetic. The magnetic behavior reported in this work for the Fe30Mn foams obtained at a N_2_ flow rate of 350 and 650 L/h could be of interest for a wide range of applications, such as electromagnetic absorbers, catalysts supports, thermal and acoustic insulation systems or wirelessly magnetically-guided porous objects in fluids.

## Figures and Tables

**Table 1:** Components and mass percentages of the slurry.

Components	Fe or Fe 30Mn	Binder	Distilled Water
Mass percent (%)	50	17	33

**Table 2:** Energy dispersive X-ray (EDX) composition of the foams produced from the Fe-containing slurry at a N_2_ flow of 180 L/h and from the FeMn-containing slurry at a N_2_ flow of 180, 350 and 650 L/h.

Sample	Mn (at %)	Fe (at %)	O (at %)
**Fe-O (180 L/h)**	0	39	61
**Fe-Mn-O (180 L/h)**	12	28	60
**Fe-Mn-O (350 L/h)**	16	26	58
**Fe-Mn-O (650 L/h)**	18	24	56

**Table 3:** Phase percentage and cell parameters (*a* and *c*) of the obtained foams.

Alloy	Phase		Cell Parameters (Å)	%
**Fe-O** (180 L/h)	hematite	Fe_2_O_3_	*a* = 5.036; *c* = 13.748	100
**Fe-Mn-O** (180 L/h)	hematite	Fe_2_O_3_	*a* = 5.038; *c* = 13.741	60
	bixbyite	FeMnO_3_	*a* = 9.417	31.5
	jacobsite	Fe_2_MnO_4_	*a* = 8.489	8.5
**Fe-Mn-O** (350 L/h)	hematite	Fe_2_O_3_	*a* = 5.036; *c* = 13.741	51
	jacobsite	Fe_2_MnO_4_	*a* = 8.483	49
**Fe-Mn-O** (650 L/h)	Hematite	Fe_2_O_3_	*a* = 5.036; *c* = 13.745	26
	jacobsite	Fe_2_MnO_4_	*a* = 8.5028	74
